# Effect of NEuroplasticity-Principles-based SEnsory-Rehabilitation (NEPSER) on sensori-motor recovery in stroke: study protocol for a randomized controlled trial

**DOI:** 10.1186/s42466-021-00108-1

**Published:** 2021-02-04

**Authors:** Kamal Narayan Arya, Shanta Pandian, G. G. Agarwal, Neera Chaudhary, Akshay Kumar Joshi

**Affiliations:** 1 Department of Occupational Therapy, Pandit Deendayal Upadhyaya National Institute for Persons with Physical Disabilities, 4 Vishnu Digamber Marg, New Delhi, 110002 India; 2 Department of Statistics, Lucknow University, Lucknow, India; 3 Department of Neurology, Vardhman Mahavir Medical College and Safdarjung Hospital, New Delhi, India

**Keywords:** Cerebrovascular accident, Fugl-Meyer, Hand, Proprioception, Somatosensory, Stereognosis

## Abstract

**Introduction:**

Up to 2/3rd of the stroke subjects may experience impairment in any of the somatosensory modalities such as light touch, proprioception, and stereognosis. The sensory recovery is strongly associated with the level of motor recovery. Very negligible sensory-based interventions have been developed and found to be evident in enhancing the sensory deficit and associated motor recovery. The possible factor for the ineffectiveness of these sensory interventions could be lack of the neuroscientific basis in formulation of the program. Thus, the objective of the study is to determine the effectiveness of a neuralplasticity-principles-based sensory-rehabilitation protocol on motor and sensory recovery, and disability of the post-stroke hemiparetic subjects.

**Methods:**

We propose to recruit 122 poststroke subjects in a randomized controlled, assessor blinded trial to be conducted in a rehabilitation-institute. The key eligibility criteria is age between 20 to 80 years, hemiparesis (right or left), ischemic or hemorrhagic stroke, 1 to 12 months poststroke, and impairment in any of the sensory modalities. The participants in the experimental group will receive **NE**uroplasticity-**P**rinciples-based **SE**nsory-**R**ehabilitation (**NEPSER**) protocol comprising active, repetitive, and meaningful training of the specific sensory modalities utilizing visuo-perceptual, cognitive, motor, and functional tasks will be imparted for 8 weeks, 5 sessions / week, each of 2 h. The control subjects will undergo only standard rehabilitation based on neurophysiological, biomechanical, and rehabilitative approaches. All the participants will be assessed for motor (Fugl-Meyer assessment, upper extremity section) and sensory recovery [Nottingham Sensory assessment (Erasmus MC modification of the revised version)] at baseline, 8-week, and 12-week follow-up. The Semmes weinstein monofilament, two-point discrimination test and modified rankin scale (disability) will be applied as secondary measures. A repeated-measures 2-way ANOVA will be used to estimate difference for the post intervention and follow-up scores between the groups.

**Perspective:**

The proposed study will lead to development of a novel rehabilitation protocol that will not only enhance the sensory recovery but also the motor and functional recovery. This may reduce the impact of stroke disability and enhance the quality of life.

**Trial registration:**

The trial has been registered under Clinical Trial Registry of India (CTRI) as CTRI/2019/09/021442 on 30th September 2019.

## Introduction

In stroke, motor paresis being a major manifestation, has always been emphasized upon. However, subtle somatosensory impairments such as reduced ability of light touch, proprioception, stereognosis, and 2-point discrimination may also be exhibited among the poststroke subjects. Up to 2/3rd of the stroke subjects may experience impairment in any of the somatosensory modalities [1, 2].

The sensory deficits hamper the ability to utilize the available motor level in the functional tasks. The sensory recovery has been found to be strongly associated with the level of motor recovery. The impaired sensation along with the complex motor deficits increases the disability manifold. For instance, an individual with diminished light touch in hand despite good finger control may not be able to manipulate an object for functional performance. The sensory impairment is also associated with the participation restriction leading to poor quality of life [3]. Thus, integration of sensory abilities is crucial for the motor recovery.

In poststroke, sensory training of varied range has been investigated [4, 5]. The techniques may range from passive techniques such as somatosensory electrical stimulation to active retraining of stereognosis, proprioception, discriminating and localizing sensations However, most of them have not exhibited sufficient evidence for their effectiveness. Additionally, most of these techniques were not utilizing the theoretical foundation of neuroplasticity. Very few studies utilized the sensori-motor training, fulfilling some of the principles of neuroplasticity. Diego et al (2013) [6] investigated the effect of sensorimotor therapy in form of constraint induced movement therapy on chronic stroke. The construct of program had components of passive motor therapy, proprioception and touch inputs along with functional training. However, the chronic subjects of 4 to 5 years and only 16 h of therapeutic sessions were not favourable for the biological recovery [7]. Carey et al (2011) [8] conducted a randomized controlled trial by providing somatosensory discrimination training for texture and object recognition, and proprioception. The investigation exhibited favourable change for sensory recovery. However, the duration was only for 10 h with a low frequency of 3 h/week as well as the impact on the motor recovery was not examined.

In relation to stroke rehabilitation, task-specificity, environment, repetition, frequency, intensity, and meaningfulness of movements or activities are some of the evident neuroplastic principles [7]. Sensory rehabilitation protocols utilizing majority of these principles are sparse. Thus, there is a need for development and testing of a novel sensory rehabilitation regime based on the concepts of neural-reorganization.

## Methods

### Aim of the trial

The initial objective of the proposed study was to develop a sensory-deficit specific rehabilitation intervention considering the motor level of the stroke subject. The development and feasibility of the protocol has been achieved. The subsequent aim is to determine the effectiveness of the protocol on motor and sensory recovery of the upper limb among the poststroke hemiparetic participants. The research question, how the sensory deficit of the upper limb interacts with the motor-recovery process and motor therapy will be explored. The sensory deficits for the upper limb or hand such as light touch, pressure, proprioception, and stereognosis will be focussed. The substantial frequency and intensity of intervention, stage- and chronicity-specific program will be the other key aspects in the intervention regime. The motor recovery in terms of voluntary motor control for the upper limb, wrist and hand will be evaluated. Further, the overall disability status of the subjects will also be assessed. The ultimate aim of this trial is to systematically integrate sensory intervention into the motor rehabilitation of the stroke subjects in order to enhance substantial motor recovery.

### Study description and study design

#### Design

Randomized controlled, assessor blinded trial.

#### Sample size

The power calculation has been conducted using the values for motor and sensory assessment from the two different studies [9, 10]. Considering the beta = 0.1 and α = 0.05, the calculation inferred that 53 subjects in each group would be sufficient to detect the desired change. However, to compensate possible drop outs (15%), 61 subjects in each group will be enrolled. Thus, the total sample size for the proposed investigation will be 122.

#### Allocation

The subjects will be randomly allocated between the experimental and control groups in a block of 10. The randomization process will be conducted by an office staff, not associated with the study, using the SPSS Version 23 software. The intervention will be allocated in the ratio of 1:1. The assignment will be serially arranged in the coded digital files. The principal investigator (K.N.A.) will enroll the potential participants and assign them into the respective group. The assessor will be unaware about the group allocation of the subjects.

#### Participant timelines

**Fig. 1:**
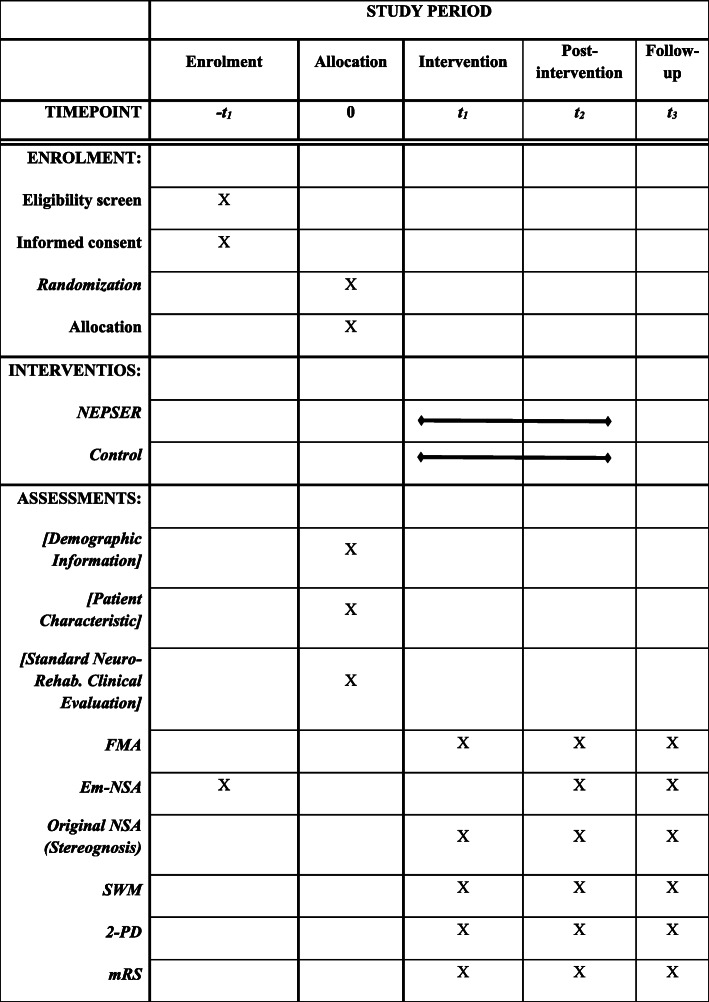
Timeline for schedule of enrolment, interventions, and assessment as per SPIRIT guidelines. -t1 = prior to enrolment, t1 = baseline assessment, t2 = assessment after 8 weeks of intervention, t3 = assessment after four-week follow-up (12 weeks after start of intervention). FMA = Fugl-Meyer assessment, Em-NSA = Erasmus MC modification of the revised Nottingham Sensory Assessment, NSA = Nottingham Sensory Assessment, SWM = Semmes-Weinstein monofilaments, 2-PD = Two-point discrimination, mRS = modified Rankin scale. NEPSER = **NE**uroplasticity-**P**rinciples-based **SE**nsory-**R**ehabilitation

The timeline for schedule of enrolment, interventions, and assessment as per SPIRIT guidelines is provided in Fig. 1*.*

### Eligibility criteria

The subjects will be recruited if they exhibit the following key criteria: 1) Age - 20 to 80 years, 2) Hemiparesis (right or left) (as assessed by Fugl-Meyer assessment [11] upper extremity subsection: 0 to 66), 3) First episode of unilateral stroke, 4) Ischemic or hemorrhagic stroke, 5) 1 to 12 months after the stroke onset, 6) Impaired or more sensory deficit of any of the sensory modalities (< 7/8) [1] as discerned by Nottingham Sensory Assessment (Erasmus MC modification of the revised version) [12], and 6) Able to comprehend instructions and perceive assessments. However, the participants will be excluded if they demonstrate any of the following: 1) Receptive communication or other language disorder (which could interfere with the assessment and treatment process), 2) Contractures and deformities of hand / finger, 3) Complex regional pain syndrome, 4) Severe cognitive or perceptual deficit (as evaluated by the National Institutes of Health Stroke Subscales [13] and clinical tests: copying and drawing, line-bisection, cancellation tasks, and functional performance), 5) Diabetic or any other neuropathy, 6) Skin disorder, and 7) Peripheral nerve injury of either of the upper limbs.

### Arms and interventions

#### Experimental group intervention

**NE**uroplasticity-**P**rinciples-based **SE**nsory-**R**ehabilitation (**NEPSER**) Protocol is based on the key principles of neuroplasticity [7, 14] established in relation to the rehabilitation of stroke subjects. In order to enhance the impaired sensory deficit, active training of the specific sensory modalities will be imparted. Similar to the motor rehabilitation regimes, the repetitiveness of sensory experiences will be provided by adequate training time. The sensory training will be imparted by utilizing visuo-perceptual, cognitive, motor, and functional tasks. Variety of meaningful tasks of daily life having sensory demand in addition to the motor will also be incorporated.

The following guidelines will be utilized while executing the experimental intervention:

- Maximum duration of the protocol will be 120 min with 20–25 min for each sensory modality with maximum of 5 min for each level of intervention and intermittent rest period. In case, there is no impairment of a certain modality, the training time for that modality will be equally distributed to other impaired ones, with a maximum of 40 min for each.
- The order of modality training will be in random order in each session.
- The training will be imparted in order of lower level of therapy to the higher ones.
- Sharp sensory stimulus will be in safer limits.
- Mirror therapy (MT) activities will be graded from unilateral (non-paretic limb performance) to bimanual (simultaneously moving on both sides).
- MT protocol will be provided as per the published study (Arya et al. 2018) [15]
- Sensory imagination (2 min) for each stimulus will be provided to prior to every sub-session of training.
- Other activities will be graded from bilateral to unilateral (constraining the non-paretic limb).
- Gradation of activities will also be done from visual feedback to vision occluded.
- Variety of sensori-motor objects / activities should be utilized for imparting the intervention.
- Auditory cues, guidance, motivation will be provided by the therapist as per the requirement.
- The constraint will be provided using a sling for sensory-functional training for higher stage patients.
- Therapy for different type of sensory deficits will be provided separately.
- Therapy for sensory deficit will be provided as per the motor recovery stage of the hand.
- Levels of intervention will range from neural activation using mirror therapy and sensory imagination to active perception of stimuli and sensory motor usage in non-functional to meaningful functional items.
- Therapy will be provided in distraction free and calm environment.
- Blindfolding the subject during the therapy session will be performed as per the need.
- The therapy may be modulated as per the extent of sensory deficit of specific type.
- Two minutes rest break after training of each modality will be provided
- Variety of items with similar texture / therapeutic value will be used.
- Home program comprising of functional touch of variety of meaningful objects/ sensation will be provided throughout the day for 2–3 h during the entire 8-week.
- The home program will be supervised through a log book maintained by patient/care taker regarding the sensory-functional usage.

Levels of therapy:

The NEPSER protocol will be imparted in a sequence of 4 levels, ranging from neural activation to functional usage of different sensory modalities.

- Level I: Neural Activation
  - Mirror therapy (illusion of the normal sensory perception using mirror-box therapy);
  - Sensory imagery (imagery of the normal sensory perception)
  - These techniques may enhance the neural activation of the sensory cortex. The activation will provide a reasonable base for active sensory training. The perception such as various texture, size, shape, and objects will be used.
- Level II: Active Sensory-Training
  - The active sensory training for modalities such as tactile awareness and localization, texture recognition, and stereognosis will be provided by prolong-duration, meaningful and bimanual sensory perception.
- Level III: Sensori-Motor therapy
  - Motor therapy will be provided using specific stimuli (textured, shape) during activities.
- Level IV: Sensory-Functional therapy
  - Prolong-duration functional therapy using various meaningful shapes, sizes and textured objects of daily use will be provided.

The detail of execution of the experimental intervention is provided in Table 1. The regime will be imparted for 40 sessions, each 2 h, 5 sessions / week across 2 months. The assessments will be carried out at baseline, 8-week post-intervention and 4-week follow-up. During the follow-up duration, the subject will receive standard rehabilitation program.

**Table 1:** Experimental intervention as per the type of sensory impairment and motor recovery stage of the hand

Sensory Impairment	Level of therapy	Motor Recovery Stage			
		BRS-H 1 & 2	BRS-H 3	BRS-H 4	BRS-H 5 & 6
**Light touch**	**Level-I**	MT (unilateral) – cotton / wool /soft toy	MT (bimanual) – cotton / wool /soft toy	MT (bimanual) – grasp-release, lateral prehension-release using cotton / wool /soft toy	MT (bimanual) – grasp and release, prehensile activity using cotton / wool /soft toy
	**Level-II**	Bilateral and unilateral perception of cotton / wool / soft toy (therapist applied)	Bilateral and unilateral perception of cotton / wool / soft toy	Bilateral and unilateral perception of cotton / wool / soft toy by grasp-release, lateral prehension and release	Bilateral and unilateral perception of cotton / wool / soft toy by prehensile activity
	**Level-III**	Bilateral movements of upper limb joints holding cotton /wool /soft toy	Unilateral movements of upper limb joints holding cotton /wool /soft toy	Unilateral perception of cotton / wool / soft toy by grasp-release, lateral prehension and release	Unilateral movement (constraining the non-paretic limb) for prehensile activity using cotton / wool /soft toy
	**Level-IV**	Bilateral functional tasks – lifting cloth / pillow	Unilateral functional tasks – lifting cloth / pillow	Unilateral functional tasks – grasp-release, lateral prehension and release of cloth / pillow	Unilateral functional tasks (constraining the non-paretic limb) – grasp-release, lateral prehension and release of cloth / pillow
**Pressure**	**Level-I**	MT (unilateral) - clay (graded resistance) activity, ball squeezing	MT (bimanual) - clay (graded resistance) activity, ball squeezing	MT (bimanual) – grasp-release, lateral prehension and release of - clay (graded resistance) activity, ball	MT (bimanual) – pinching clay (graded resistance) activity
	**Level-II**	Bilateral and unilateral perception of clay (graded resistance) activity, ball squeezing (therapist applied)	Bilateral and unilateral perception of clay (graded resistance) activity, ball squeezing	Bilateral and unilateral perception of– grasp-release, lateral prehension and release - clay (graded resistance) activity, ball	Bilateral and unilateral perception of pinching clay (graded resistance) activity
	**Level-III**	Bilateral movements of upper limb joints holding high- density foam cylindrical blocks	Unilateral movements of upper limb joints holding high- density foam cylindrical blocks	Unilateral movements of grasp-release, lateral prehension and release - high- density foam blocks	Unilateral movement (constraining the non-paretic limb) for pinching clay (graded resistance) activity
	**Level-IV**	Bilateral movements of upper limb joints holding textured water glass/bottle	Uilateral movements of upper limb joints holding textured water glass/bottle	Unilateral movements of grasp-release, lateral prehension and release - textured water glass/bottle, key, cardboard	Unilateral functional tasks (constraining the non-paretic limb) – pinching and picking grains/coins
**Pin prick**	**Level-I**	MT (unilateral)– Scrubber / Steel wool/Velcro (rough)	MT (bimanual) – Scrubber / Steel wool/Velcro (rough)	MT (bimanual)– grasp-release, lateral prehension and release of Scrubber / Steel wool/Velcro (rough)	MT (bimanual)– prehensile activity using Scrubber / Steel wool/Velcro (rough
	**Level-II**	Bilateral and unilateral perception of Scrubber / Steel wool/Velcro (therapist applied)	Bilateral and unilateral perception of Scrubber / Steel wool/Velcro	Unilateral perception of grasp-release, lateral prehension and release of Scrubber / Steel wool/Velcro	Unilateral perception of prehensile activity of items textured with Scrubber / Steel wool/Velcro
	**Level-III**	Bilateral movements of upper limb joints holding textured (Scrubber / Steel wool/Velcro) cylindrical blocks	Unilateral movements of upper limb joints holding textured (Scrubber / Steel wool/Velcro) cylindrical blocks	Unilateral movements of upper limb joints comprises grasp-release, lateral prehension and release of textured (Scrubber / Steel wool/Velcro) blocks	Unilateral movements (constraining the non-paretic limb) of upper limb joints comprises prehensile activity of items textured with Scrubber / Steel wool/Velcro
	**Level-IV**	Bilateral movements of upper limb joints holding textured (Scrubber / Steel wool/Velcro) water glass/bottle	Unilateral movements of upper limb joints holding textured (Scrubber / Steel wool/Velcro) water glass/bottle	Unilateral movements of grasp-release, lateral prehension and release of textured (Scrubber / Steel wool/Velcro) daily use items	Unilateral movement (constraining the non-paretic limb) of prehensile activity of daily use items textured with Scrubber / Steel wool/Velcro
**Sharp-blunt discrimination**	**Level-I**	MT (unilateral)- Dellon’s Discriminator disc (therapist applied)	MT (bimanual) - Dellon’s Discriminator disc (therapist applied)	MT (bimanual) – sharp and blunt textured items alternatively	MT (bimanual) – prehensile activity of sharp and blunt textured items
	**Level-II**	Bilateral and unilateral sharp and blunt discrimination (therapist applied)	Bilateral and unilateral sharp and blunt discrimination	Unilateral sharp and blunt discrimination	Unilateral prehensile activity of sharp and blunt textured items
	**Level-III**	Bilateral movements of upper limb joints holding dual spikes/textured (blunt and sharp) cylindrical blocks	Unilateral movements of upper limb joints holding dual spikes/textured (blunt and sharp) cylindrical blocks	Unilateral movements of upper limb joints comprises sharp and blunt textured items along with manipulation; grasp-release, lateral prehension and release	Unilateral movements (constraining the non-paretic limb) of upper limb joints comprises prehensile activity of sharp and blunt textured items
	**Level-IV**	Bilateral movements of upper limb joints holding dual spikes/textured (blunt and sharp) water glass/bottle	Unilateral movements of upper limb joints holding dual spikes/textured (blunt and sharp) water glass/bottle	Unilateral sharp and blunt textured daily use items manipulation; grasp-release, lateral prehension and release	Unilateral movements (constraining the non-paretic limb) of upper limb joints comprises prehensile activity of sharp and blunt textured daily use items
**Proprioception**	**Level-I**	MT (unilateral)- task-based movements of shoulder, elbow, wrist; Movement of non-paretic upper limb and perceiving the limb position and movement with vision occluded.	MT (bimanual) - task-based movements of shoulder, elbow, wrist; Movement of the upper limbs and perceiving the limb position and movement with vision occluded.	MT (bimanual) - task-based movements of shoulder, elbow, wrist, hand, thumb and fingers; Movement of the joints and perceiving the limb position and movement with vision occluded.	MT (bimanual) - task-based movements of all upper limb especially fingers; Movement of the joints and perceiving the finger position and movement with vision occluded.
	**Level-II**	Bilateral movements of shoulder, elbow, wrist with visual feedback followed by vision occluded	Unilateral movements of shoulder, elbow, wrist with visual feedback followed by vision occluded	Unilateral movements of shoulder, elbow, wrist hand, thumb and fingers; with visual feedback followed by vision occluded	Unilateral movements (constraining the non-paretic limb) of all upper limb especially fingers; with visual feedback followed by vision occluded
	**Level-III**	Bilateral activity-based movements of shoulder, elbow, wrist with visual feedback followed by vision occluded	Unilateral activity-based movements of shoulder, elbow, wrist with visual feedback followed by vision occluded	Unilateral activity-based movements of shoulder, elbow, wrist hand, thumb and fingers; with visual feedback followed by vision occluded	Unilateral activity based movements (constraining the non-paretic limb) of all upper limb especially fingers; with visual feedback followed by vision occluded
	**Level-IV**	Bilateral functional task-based movements of shoulder, elbow, wrist with visual feedback followed by vision occluded	Unilateral functional task-based movements of shoulder, elbow, wrist with visual feedback followed by vision occluded	Unilateral functional task-based movements of shoulder, elbow, wrist, hand, thumb and fingers; with visual feedback followed by vision occluded	Unilateral functional task-based movements (constraining the non-paretic limb) of all upper limb especially fingers; with visual feedback followed by vision occluded
**Stereognosis**	**Level-I**	MT (unilateral) – 10 objects	MT (bimanual) – 10 objects; similar objects on both the sides.	MT (bimanual) – 10 objects; similar objects on both the sides; manipulation of objects by grasp-release, lateral prehension and release	MT (bimanual) – 10 small objects such as beads; similar objects on both the sides along with the manipulation of objects
	**Level-II**	Perceiving the 10 objects bilaterally with visual feedback followed by vision occluded	Perceiving the 10 objects unilaterally with visual feedback followed by vision occluded	Perceiving the 10 objects unilaterally with visual feedback followed by vision occluded; manipulation of objects by grasp-release, lateral prehension and release	Perceiving the 10 small objects unilaterally with visual feedback followed by vision occluded
	**Level-III**	Bilateral manipulation (reaching, lifting, placing) of 10 objects with visual feedback followed by vision occluded	Unilateral manipulation (reaching, lifting, placing) of 10 objects with visual feedback followed by vision occluded	Unilateral manipulation (reaching, lifting, placing, grasp-release, lateral prehension and release) of 10 objects (mixed together) with visual feedback followed by vision occluded	Unilateral manipulation (reaching, lifting, placing, in-hand manipulation) of 10 small objects (mixed together) with visual feedback followed by vision occluded
	**Level-IV**	Bilateral manipulation (reaching, lifting, placing) of variety of daily use items with visual feedback followed by vision occluded	Unilateral manipulation (reaching, lifting, placing) of variety of daily use items with visual feedback followed by vision occluded	Unilateral manipulation (reaching, lifting, placing, grasp-release, lateral prehension and release) of 10 daily use items (mixed together) with visual feedback followed by vision occluded	Unilateral manipulation (reaching, lifting, placing, in-hand manipulation) of 10 small daily use objects (mixed together) with visual feedback followed by vision occluded

*BRS-H* Brunnstrom Recovery Stage – Hand, *MT* Mirror therapy

#### Control group intervention

The control group will be provided same duration of standard rehabilitation program. The intervention will comprise of mirror therapy (movement based), motor therapy for the upper limb (utilizing activities such as pegs, blocks, pyramids, ball, and simulated sanding), and constraint–induced movement therapy. All the maneuvers will primarily comprise of motor tasks.

#### Post trial care

After the completion of the study protocol, all the enrolled subjects will further receive conventional rehabilitation services.

### Outcome measures

#### Primary measures

##### Fugl-Meyer assessment

Considering the motor paresis as one of the major manifestation of stroke and intense desire of stroke survivors to regain motor ability, the Fugl-Meyer assessment [11] (FMA) has been selected as a primary measure for the present study. FMA, a performance-based motor measure has five sections to examine motor, balance, sensation, range of motion and pain. The upper extremity motor section of FMA (FMA-UE) will be utilized in the investigation as a primary measure. The items of FMA-UE are hierarchically organized to quantify motor control in terms of reflexive, synergistic, mixed-synergistic, beyond-synergistic, coordinated movements. Specifically, FMA-UE is divided into 4 categories: upper extremity (18 items), wrist (5 items), hand (7 items), and coordination / speed (3 items). All items of each category are scored on a 3-point ordinal scale; ranging from 0 (no performance) to 2 (complete performance). FMA-UE is scored out of 66, with sub-score of 36 for the upper arm (FMA-UA) and 30 for the wrist and hand (FMA-WH). The mean FMA-UE scores (including FMA-UA and FMA-WH) at baseline, after 8-week intervention and 4-week follow-up will be considered for the analysis. FMA is a reliable and valid measure as well as most preferred for the poststroke motor related studies.

##### Nottingham sensory assessment (Erasmus MC modification of the revised version)

In view of the other primary objective, the somatosensory impairment will be assessed by the Erasmus MC modification of the revised Nottingham Sensory Assessment (Em-NSA). The measure comprises 5 somatosensory deficits to be assessed in the upper limb, namely light touch, pressure, pinprick, sharp-blunt discrimination, and proprioception. The deficits will be assessed as per the standard guidelines provided by the developers [12]. Each deficit is measured for the upper arm, forearm, hand and fingers, with a score range from 0 (absent) to 2 (no impairment) for every body part. The subtotal for each modality will be 8 with a total score for the Em-NSA as 40. The Em-NSA has demonstarted good to excellent intrarater and interrater reliability and validity and is a recommended and commonly used measure of sensory deficit in major neurological diseases including stroke [12].

Stereognosis will be assessed by the original NSA that comprises identification of 11 daily used items touch and manipulation in absence of vision. The assistance of the assessors in form of item manipulation will be imparted whenever required. Each item is score on 2-point rating (0 = absent to 2 = normal). The total stereognosis score range from 0 to 22. A score of 19/22 indicates impaired stereognosis. The stereognosis section of the NSA exhibited a moderate to good test-retest reliability among stroke subjects [1, 16]. Similar to the FMA, the analysis will be conducted for mean scores of total Em-NSA (including each sensory modality) and NSA (stereognosis) at postintervention and follow-up.

#### Secondary measures

##### Semmes weinstein monofilament

Semmes-Weinstein monofilaments (SWM), standardized nylon fibres measure diminished cutaneous threshold. SWM filaments are graded to measure normal light touch, diminished light touch, diminished protective sensation, and loss of protective sensation. The filaments will be applied on palm (thenar and hypothenar areas) and fingers (tips and phalanges) of the upper limb to score the perceived response for the most sensitive to filaments. The cutaneous threshold is indicated in form of force ranging from 0.0045 (normal light touch) to 447 g (loss of protective sensation). SWM is considered to be a standard measure of assessing cutaneous threshold [17]. Further, it has been used in various stroke studies to measure the sensory deficits [15].

##### Two-point discrimination test

The two-point discrimination (2PD) test will be used to examine discriminative acuity, subject’s ability to perceive the two points being touched simultaneously. The static 2PD test using standard aethesiometer will be carried out in the palm and fingers of both the hands. The scoring will be noted in mm for the least distance between the perceived two points. The lesser the score the more will be the tactile acuity. In the proposed study, 2-PD measurement will be performed for the thenar and hypothenar areas (palm) and tips and phalanges (fingers). 2-PD is considered to be a reliable measure for stroke subjects [18].

##### Modified Rankin scale

The modified Rankin scale (mRS) [19], a universally used measure of disability will be utilized to evaluate the disability outcome of the NEPSER intervention in comparison to the standard rehabilitation regime. mRS scoring will be performed on ordinal scale range from 0 (no symptoms at all) to 5 (severe disability). mRS, a reliable and valid measure preferred to be an end point measure for stroke-related trials of new interventions.

#### Statistical methods

The data will be analyzed by using IBM SPSS version 23.0. The demographic and baseline features of the study participants will be analyzed in form of mean (SD) / median (IQR) / n (%) and appropriate test [(Mann-Whitney U (U) / independent t (t) / chi-square (χ^2^)] tests will be used to analyze the difference for the characteristics between the groups. For inferential statistics, an intention-to-treat analysis method will be used by carrying forward the last observation for the missing data. A repeated-measures 2-way ANOVA (continuous data; within factor, time; between factor, group) will be used to estimate difference for the post intervention and follow-up scores between the groups. The pre-intervention score will be considered as the covariate with group as the independent variable and post-intervention or follow-up score as the dependent variable. The significance level will be set at *P* < .05.

### Contacts

This single-centre study is proposed to be conducted in the Neuro-Rehab laboratory of the Department of Occupational therapy, Pt. Deendayal Upadhyaya National Institute for Persons with Physical Disabilities, New Delhi, India. The study is funded by Indian Council of Medical Research, New Delhi, India.

### Perspective

The proposed study will lead to development, a novel rehabilitation protocol for the management of sensory-motor deficits in stroke. NEPSER trial represents the role of somatosensory training based on the concepts of neuroplasticity; in terms of key principles and application manoeuvre. The crucial components are frequency and intensity of protocol, recovery stage and chronicity of stroke, and management for specific sensory-deficit type. Most importantly, the integration of motor and sensory tasks has been systematically incorporated into the sensory protocol. It is hypothesized that the NEPSER regime will not only enhance the sensory recovery but also the motor and functional recovery. The improved sensation and motor activity may allow the subjects to utilize the paretic upper limb in daily performances. This may reduce the impact of stroke disability and enhance the quality of life.

The recruitment for the study has been started from October 2019 and expected to be completed by July 2022. The data from the proposed investigation will also provide the information about the various types of sensory deficits and their interaction with the motor recovery. In addition to this, the study findings will provide directions to consider sensory aspects in poststroke motor rehabilitation.

In view of various factors associated with the stroke, some of possible limitations of this study could be heterogeneity of the potential study subjects in terms of area of brain involvement, modality of sensory deficit, and motor recovery stages.

## Data Availability

Not applicable.

## Abbreviations

BRS-H: Brunnstrom Recovery Stage – Hand
Em-NSA: Erasmus MC modification of the revised Nottingham Sensory Assessment
FMA: Fugl-Meyer Assessment
mRS: Modified Rankin scale
MT: Mirror therapy
NEPSER: **NE**uroplasticity-**P**rinciples-based **SE**nsory-**R**ehabilitation
NSA: Nottingham Sensory Assessment
SPIRIT: Standard Protocol Items: Recommendations for Interventional Trials
SWM: Semmes-Weinstein monofilaments
UE: Upper extremity
2PD: Two-point discrimination test
